# Pro-Aging Effects of Glucose Signaling through a G Protein-Coupled Glucose Receptor in Fission Yeast

**DOI:** 10.1371/journal.pgen.1000408

**Published:** 2009-03-06

**Authors:** Antoine E. Roux, Alexandre Leroux, Manal A. Alaamery, Charles S. Hoffman, Pascal Chartrand, Gerardo Ferbeyre, Luis A. Rokeach

**Affiliations:** 1 Department of Biochemistry, Université de Montréal, Montréal, Québec, Canada; 2 Biology Department, Boston College, Chestnut Hill, Massachusetts, United States of America; Washington University School of Medicine, United States of America

## Abstract

Glucose is the preferred carbon and energy source in prokaryotes, unicellular eukaryotes, and metazoans. However, excess of glucose has been associated with several diseases, including diabetes and the less understood process of aging. On the contrary, limiting glucose (i.e., calorie restriction) slows aging and age-related diseases in most species. Understanding the mechanism by which glucose limits life span is therefore important for any attempt to control aging and age-related diseases. Here, we use the yeast *Schizosaccharomyces pombe* as a model to study the regulation of chronological life span by glucose. Growth of *S. pombe* at a reduced concentration of glucose increased life span and oxidative stress resistance as reported before for many other organisms. Surprisingly, loss of the Git3 glucose receptor, a G protein-coupled receptor, also increased life span in conditions where glucose consumption was not affected. These results suggest a role for glucose-signaling pathways in life span regulation. In agreement, constitutive activation of the Gα subunit acting downstream of Git3 accelerated aging in *S. pombe* and inhibited the effects of calorie restriction. A similar pro-aging effect of glucose was documented in mutants of hexokinase, which cannot metabolize glucose and, therefore, are exposed to constitutive glucose signaling. The pro-aging effect of glucose signaling on life span correlated with an increase in reactive oxygen species and a decrease in oxidative stress resistance and respiration rate. Likewise, the anti-aging effect of both calorie restriction and the *Δgit3* mutation was accompanied by increased respiration and lower reactive oxygen species production. Altogether, our data suggest an important role for glucose signaling through the Git3/PKA pathway to regulate *S. pombe* life span.

## Author Summary

Lowering caloric intake by limiting glucose (the preferred carbon and energy source) increases life span in various species. Excess glucose can have deleterious effects, but it is not clear whether this is due to the caloric contribution of glucose or to some other effect. Glucose sensed by the cells activates signaling pathways that, in yeast, favor the metabolic machinery that makes energy (glycolysis) and cell growth. The sensing of glucose also reduces stress resistance and the ability to live long. Does glucose provoke a pro-aging effect as a result of its metabolic activity or by activating signaling pathways? Here we addressed this question by studying the role of a glucose-signaling pathway in the life span of the fission yeast *S. pombe*. Genetic inactivation of the glucose-signaling pathway prolonged life span in this yeast, while its constitutive activation shortened it and blocked the longevity effects of calorie restriction. The pro-aging effects of glucose signaling correlated with a decrease in mitochondrial respiration and an increase in reactive oxygen species production. Moreover, a strain without glucose metabolism is still sensitive to detrimental effects of glucose due to signaling. Our work shows that glucose signaling through the glucose receptor *GIT3* constitutes the main cause responsible for the pro-aging effects of glucose in fission yeast.

## Introduction

Glucose is the major carbon source entering the metabolic pathways. Glucose ultimately generates ATP to supply the energy necessary for the cell biosynthetic and functional demands. Substantial evidences support the idea that excess glucose acts as a pro-aging and pathogenic factor [1],[2]. Consistently, lowering glucose intake in a calorie restriction diet increases life span in many species, from yeasts to mammals [3],[4].

Research carried out in *Saccharomyces cerevisiae* has been fruitful to unravel the role of nutrient sensing in longevity. Mutations blocking the action of genes controlling nutrient- signaling pathways increase replicative life span (RLS), defined as the number of times a mother yeast cell produces a daughter cell [5]–[10]. For instance, genetic deletion of PKA signaling via Gpr1 or Gpa2 genes resulted in the extension of RLS [11]. Likewise, nutrient-signaling shortens chronological life span (CLS), the time a yeast population remains viable in stationary phase [12]–[15]. In other words, nutrient-signaling pathways have a pro-aging effect in budding yeast. So far, mutations found to increase life span in *S. cerevisiae* map to genes that respond to multiple nutrients, such as the PKA, Sch9 and Tor pathways [16]. Glucose is the major source of calories for yeast. Experimentally, calorie restriction (CR) is achieved by reducing the concentration of glucose in *S. cerevisiae* cultures. Under these conditions, yeast cells exhibit an increase in both their replicative life span, and their CLS. It is therefore possible that nutrients, and more particularly the glucose-signaling pathway, are major regulators of the effects of calorie restriction on aging.

In yeast, the connection between nutrient sensing and mitochondrial activity has been depicted in different contexts. This regulation of mitochondria allows yeast to adapt its energy metabolism to the available nutrients, and is crucial for the control of longevity [16]. Several genetic studies demonstrate that forcing *S. cerevisiae* to use respiration instead of fermentation induces a gain in both chronological and replicative life span [17]–[20]. To summarize, the activity of nutrient-signaling pathways seem to promote aging by inhibiting both stress resistance and respiration. However, the predominance and the interdependence of each of these two functions, metabolic changes and signaling in the control of longevity are still nebulous.

Our laboratory introduced *Schizosaccharomyces pombe* as a model organism for the study of chronological aging [21]. The use of this particular yeast is justified by the differences existing with budding yeast in traits that can potentially affect longevity. Both have been referred as Crabtree-positive yeast because of their capability to repress mitochondrial respiration in favour of glycolysis when glucose is abundantly available [22]. Nevertheless in fission yeast, the Crabtree effect is less pronounced than in *S. cerevisiae* since the inhibition of oxygen consumption by glucose is smaller; in other words *S. pombe* maintains a higher respiration rate in the presence of glucose [22]. Consistently, it is hard to isolate respiratory-deficient cells (*petite*) in *S. pombe* [23],[24], while these mutants occur spontaneously in *S. cerevisiae*. Furthermore, *S. pombe* differs from *S. cerevisiae* because of its lack of glyoxylate cycle that makes it inefficient in ethanol consumption as carbon source [25],[26]. Finally, fission yeast is also distinguishable in its mitochondrial inheritance which is mediated by microtubules like in higher eukaryotes [27].

In the present study, we wished to determine whether glucose metabolism or extracellular glucose signaling is responsible for the regulation of life span. We found that environmental glucose decreases CLS in *S. pombe* in a dose-dependent manner, and this effect is mimicked in cells lacking the glucose receptor Git3p, a G protein-coupled receptor (GPCR) which signals the presence of glucose in the medium through a cAMP/PKA pathway [28],[29]. Consistently, the constitutive activation of the Gα subunit of the G protein-coupled to the glucose receptor significantly decreases CLS. Deletion in the Git3/PKA signaling is characterized by higher oxidative stress defense, respiration and mitochondrial membrane potential; the same features observed in CR. Interestingly, CR has no effect either on stress defense or longevity in the strain constitutively activated for Git3/PKA (by mutational activation of the Gα subunit), although it still enhances respiration. Knockout of *S. pombe* hexokinase genes (*hxk1* and *hxk2*), which are required to channel extracellular glucose into glycolysis, does not extend CLS in *S. pombe*. On the contrary, these mutant yeast strains accumulated glucose in the medium, exhibited increased glucose signaling and accelerated aging. Reduction of extracellular glucose or mutation of the glucose receptor Git3p rescued their aging phenotype. Altogether, our data suggest that glucose signaling constitutes the main pathway in the pro-aging effect of glucose in fission yeast.

## Results

### Growth on Glucose Decreases Chronological Life Span in Fission Yeast

**Figure 1:**
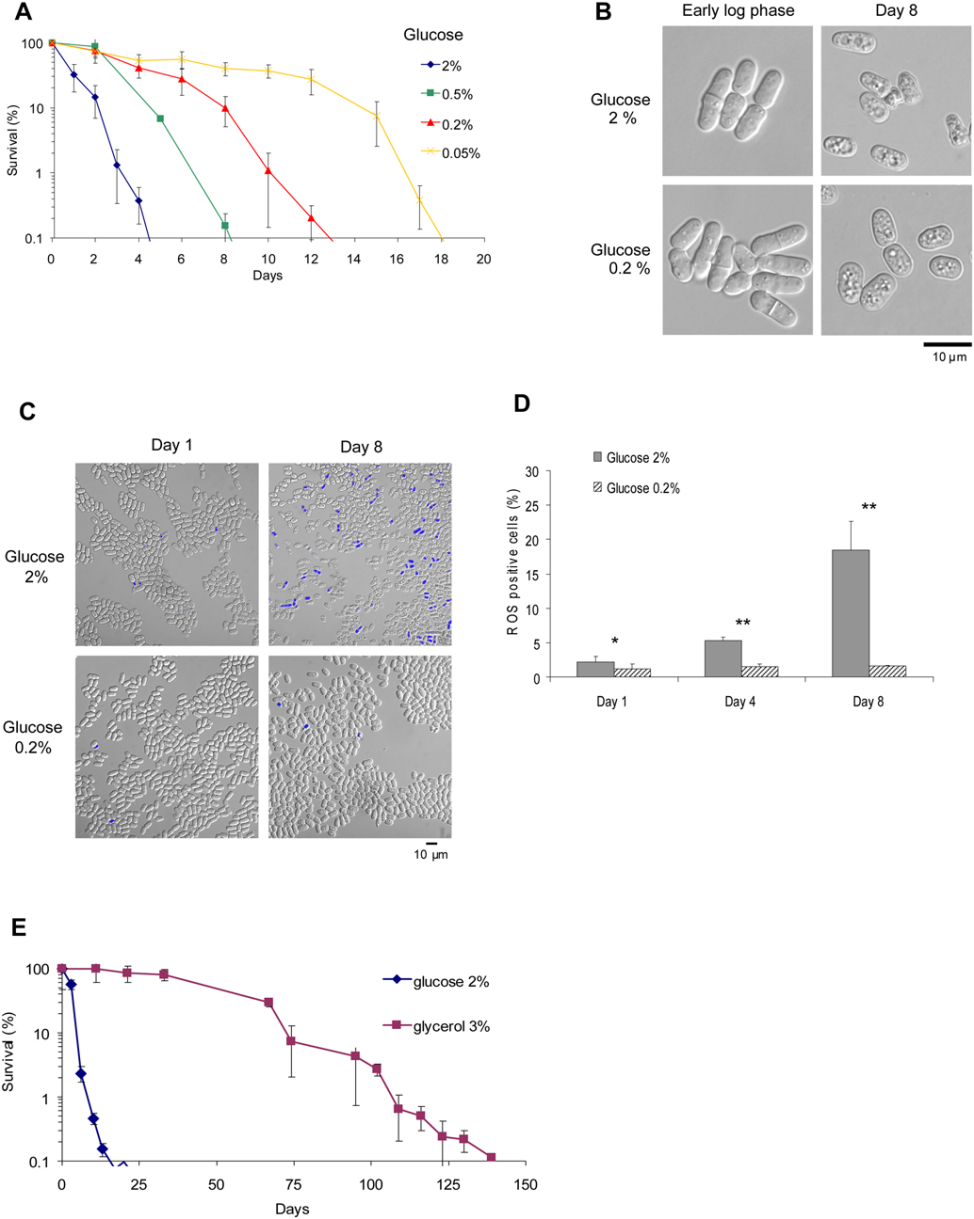
Pro-aging effects of glucose in *S. pombe*. (A) Chronological life span of a wild type strain measured at different glucose concentrations in YEC by colony forming unit (CFU) counting. Percent on the graphs refers to the concentration of glucose. Survival (Y axis) is expressed as the ratio of the number of colonies at a given time-point on the number of colonies at the beginning of stationary phase (100%). (B) Morphology of wild type cells at glucose 2% and 0.2% observed under microscope by Nomarski at early exponential phase and stationary phase (day 8). (C) ROS measurements by DHR 123 staining in WT cells grown in 2% and 0.2% glucose. Yeast cells were stained at days 1 and 8 of the stationary phase, and analysed under microscope. (D) Quantitation of ROS-positive cells in WT cells grown in 2% and 0.2% glucose. Yeast cells were stained at days 1, 4 and 8 of the stationary phase and counted under microscope. Data shown are mean±standard deviation of the mean of three independent samples assayed. *, *p*<0.02; **, *p*<0.01, Student test, 2% glucose versus 0.2% glucose at a given day. (E) Chronological life span of wild type grown in SDC medium supplemented with glucose 2% or glycerol 3%.

To study the effects of glucose concentration on CLS, wild-type *S. pombe* cells were cultured in rich medium with different concentrations of glucose. Survival was assessed by counting colony forming units (CFU) as a function of time, after cells entered stationary phase [21]. Decreasing the concentration of glucose from 2% to 0.05% resulted in a dose-dependent extension of chronological life span (Figure 1A). Cultures with higher glucose concentration exhibited a premature appearance of aged-cell phenotype upon entering the stationary phase. This phenotype is characterized by a shrunken shape and oversized vacuoles (Figure 1B). DNA content analysis by flow cytometry revealed that cells cultured in glucose 2% and 0.2% displayed a typical G2 cell-cycle arrest in stationary phase (*data not shown*). Moreover, the cells had similar doubling times at different glucose concentrations during the exponential growth phase of the culture (Figure S1).

Aging in yeast results in part from cellular damage due to the accumulation of reactive oxygen species (ROS) [30]. In agreement, cells cultured in higher glucose concentrations accumulated more ROS than cells grown at lower glucose concentrations, as shown by staining with dihydrorhodamine 123 and dihydroethidium (Figure 1C and 1D and Figure S2). On the other hand, culturing cells in SMC medium lacking glucose and containing glycerol as carbon source increased chronological longevity up to tenfold longer than in 2% glucose (Figure 1E). Glycerol as sole carbon source forces the cell metabolism toward mitochondrial respiration, as evidenced by the diminished growth rate and the rise in oxygen consumption [31]. Altogether, these results confirm that the relationship between nutrition and longevity in *S. pombe* is similar to that observed in other model organisms.

### A GPCR-Initiated Signaling Pathway Mediates Pro-Aging Effects of Glucose in *S. pombe*

**Figure 2:**
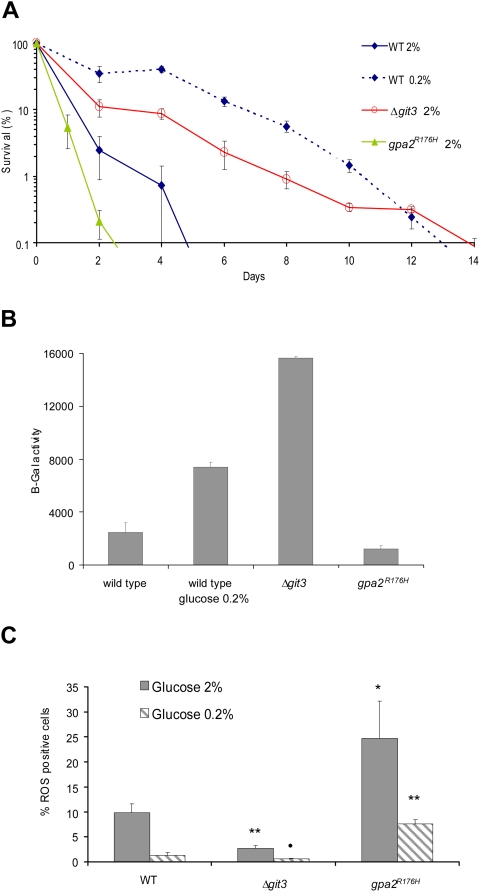
Reduction of CLS due to glucose requires signaling via the glucose receptor and G protein. (A) CLS of constitutively activated G protein mutant *gpa2^R176^* ^H^ and deletion of glucose receptor Git3p (*Δgit3*) in 2% glucose compared to wild type in 2% and 0.2% glucose. On the graphs, 2% and 0.2% refers to the concentration of glucose at the start of the culture. (B) β-Galactosidase activity of strain with *fbp1-lacZ* reporter whose expression is repressed by signaling via the Git3/PKA pathway. Cells were collected at late exponential phase and β-Gal activity was measured. (C) Accumulation of ROS-positive cells at day 4 of stationary phase in *Δgit3* and *gpa2^R176H^* grown in 2% and 0.2% glucose. Data shown are mean±standard deviation of the mean of three independent samples assayed. •, *p*<0.1; *, *p*<0.02; **, *p*<0.01, student test, WT versus mutant at the same glucose concentration.

A number of mechanisms could account for the pro-aging effects of glucose in *S. pombe*. For instance, the effect of glucose on aging could be due to extracellular glucose sensing (signaling pathway) or through intracellular glucose effects including glucose metabolism and cytoplasmic glucose sensing. To distinguish between them, we studied a strain deleted for the *git3^+^* cytoplasmic membrane glucose receptor gene. *S. pombe* cells lacking this receptor (*Δgit3*) exhibited extension of their CLS (Figure 2A), suggesting that the pro-aging effects of glucose depends, at least in part, on the activation of a signaling pathway initiated by this receptor. To further confirm this idea, we used a constitutively active Gα subunit (Gpa2^R176H^p) that acts downstream of Git3p in the glucose-signaling pathway. Gpa2^R176H^p constitutively activates the PKA kinase independently of the presence of glucose by promoting the synthesis of a high level of cAMP [28],[32]. As expected, cells expressing this activated Gα protein displayed a significantly reduced CLS (Figure 2A).

To confirm that *Δgit3* and *gpa2* ^R176H^ cells have decreased and increased glucose signaling, respectively, we took advantage of the fact that in *S. pombe*, glucose represses the transcription of the fructose-1,6-bisphosphatase *fbp1* ^+^ gene via PKA activation [33],[34]. We used an *fbp1*-driven *lacZ* reporter integrated in the *S. pombe* genome to measure *fbp1* transcription [35],^36^. Thus, the β-galactosidase activity inversely reflects the level of PKA activation in this glucose-sensing pathway. As expected, at late logarithmic phase, deletion of *git3^+^* increased expression of this reporter, while the *gpa2* ^R176H^ mutation reduced its expression (Figure 2B). Consistently, culturing WT cells in low glucose conditions also increased *fbp1-lacZ* expression (Figure 2B).

Since chronological aging in yeast is linked to the accumulation of ROS [21],[30],[37], we next measured the levels of ROS in *Δgit3* and *gpa2* ^R176H^ cells. As expected, deletion of the glucose receptor reduced ROS levels, while the constitutive activation of the glucose-signaling pathway increased ROS levels (Figure 2C). Although these results suggest that glucose signaling regulates aging independently of glucose utilization, it is possible that loss of the glucose-signaling pathway reduces glucose metabolism in this mutant. Indeed, the PKA pathway is known to control glucose intake via the regulation of hexose transporters responsible for glucose import in *S. cerevisiae* [38],[39]. We thus measured glucose consumption and found that mutations affecting the glucose-signaling pathway did not change the rate of glucose consumption (Figure S3). In conclusion, these results suggest that the glucose-signaling pathway controls chronological aging independently of glucose intake and utilization.

### Calorie Restriction and Disabled Glucose Signaling Increase Respiration

Experimentally, the intervention referred as calorie restriction (CR) is achieved by reducing the calorie intake of an organism and represents the most effective way to increase life span [3]. This phenomenon has been verified in almost all species studied, from yeast to mammals [7],[40] including non-human primates [41]. CR improves general health and delays the inception of many late-onset diseases in a variety of organisms [42].

In *S. cerevisiae*, calorie restriction is implemented by culturing the cells on low glucose concentrations [5],[15],[43]. Above, we showed that culturing *S. pombe* in low glucose decreases glucose signaling, and demonstrated that mutations affecting this signaling pathway increase the life span of *S. pombe* when cultured on high glucose concentration.

**Figure 3:**
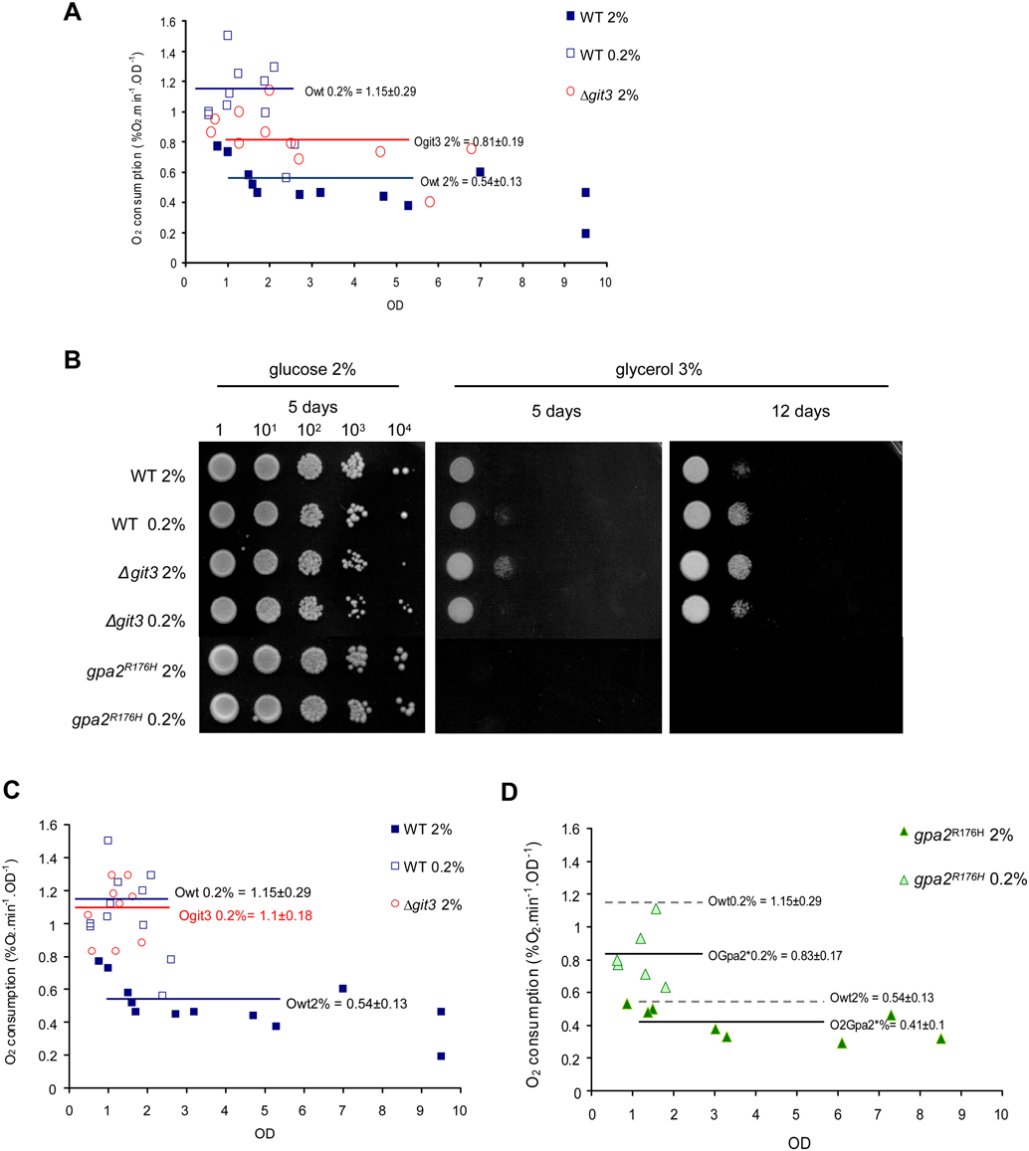
Respiration rate is increased in both CR and in strains with defects in glucose signaling. (A) Oxygen consumption measured using a Clark electrode for WT in low and high glucose, and for glucose receptor deleted strain *Δgit3*. Owt, Ogit3 represents the average of the oxygen consumption in exponential phase (calculated with OD_595_<7 at 2% glucose and OD_595_<2.1 at 0.2% glucose). (B) Growth of WT, *Δgit3* and *gpa2^R176H^* switched from fermentative glucose medium to respiratory glycerol medium. (C) Oxygen consumption of *Δgit3* at 0.2% glucose as compared to wild type. (D) Oxygen consumption for constitutively active Gα subunit (*gpa2* ^R176H^) in low and high glucose as compared to WT. Ow_t_, OGpa2* represents the average of the oxygen consumption in exponential phase (calculated with OD_595_<7 at 2% glucose and OD_595_<2.1 at 0.2% glucose).

Increased respiration correlates with longevity in yeast [17],[20], and mammals [4]. In yeast, low glucose availability leads to a switch of the pyruvate metabolism from fermentation toward mitochondrial tricarboxylic acid cycle and respiration [26],[44]. To determine if mutations in the glucose-signaling pathway affect respiration in *S. pombe*, we measured the oxygen consumption of long-lived *Δgit3* and short-lived *gpa*2^R176H^ cells. In high glucose, we observed that *Δgit3* cells display a higher level of oxygen consumption as compared to that of WT (Figure 3A). The effect of respiration on the mitochondrial membrane potential (Δψ_m_) was determined using the DiOC_6_ dye and showed that the Δψ_m_ in stationary phase cells was higher in *Δgit3* compared to WT cells (Figure S4). Interestingly, WT cells cultured in 0.2% glucose exhibited a higher level of oxygen consumption than the *Δgit3* mutant in 2% glucose (Figure 3A), and an increased mitochondrial membrane potential (Δψ_m_) in early exponential phase (Figure S4). This could explain why CR is slightly more efficient than the *git3^+^* deletion in extending CLS (Figure 2A). Higher respiration was concomitant with a better growth on respiration medium (glycerol 3%) of both, WT cells previously grown on CR conditions and *Δgit3* cells grown on either normal or CR conditions (Figure 3B). In addition to glucose repression, the participation of the PKA/cAMP-mediated signaling pathway in mitochondrial functions has been suggested in budding yeast [45],[46]. Our data and the observation that *pka1^+^* deletion increased respiration as well (*not shown*) support the involvement of Git3/PKA in the regulation of mitochondrial functions in fission yeast.

To investigate if Git3/PKA is the only pathway regulating the metabolic switch toward mitochondrial respiration during CR, we subjected *Δgit3* cells to CR and measured respiration. We observed an increase in respiration when *Δgit3* mutation was combined to CR compared to *Δgit3* cells in 2% glucose (Figure 3A and C). Hence, CR can increase respiration by mechanisms independent of the glucose receptor Git3.

On the other hand, we observed that the activated Gpa2^R176H^p prevents the full activation of respiration induced by CR (Figure 3D). These results are supported by the observation that g*pa2* ^R176H^ cells did not grow on respiration medium (glycerol) (Figure 3B). Moreover, as oxygen consumption of WT and *Δgit3* cells was 30% higher than *gpa*2^R176H^ cells in CR, we observed that the Δψ_m_ of *Δgit3* and WT cells in stationary phase remained higher than *gpa*2^R176H^ cells (Figure S4). Altogether these data suggest that CR and reduced glucose signaling are not equivalent, and that Git3/PKA is involved in the control of respiration.

### Calorie Restriction and Disabled Glucose Signaling Increase Stress Resistance

**Figure 4:**
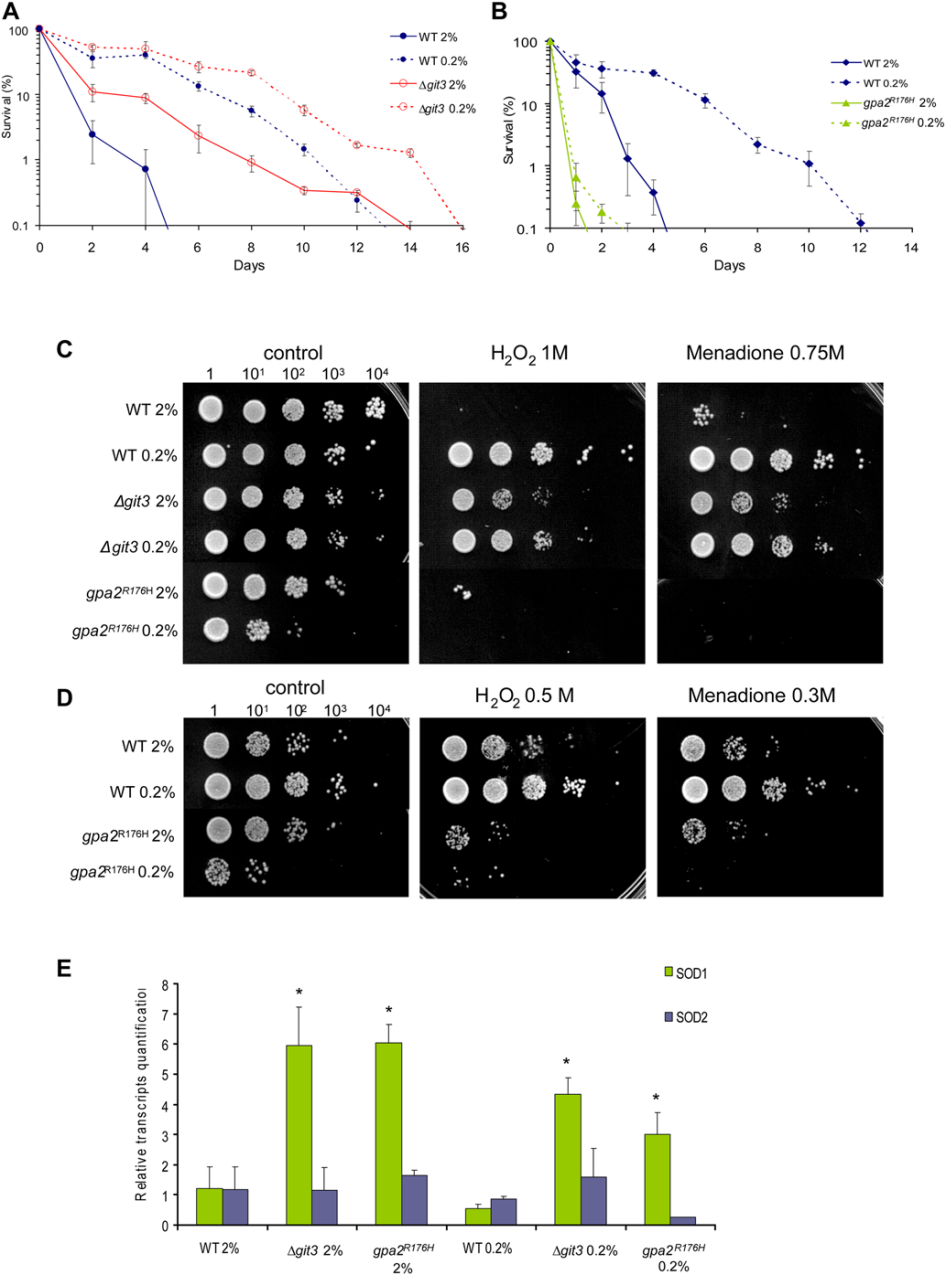
Correlation between longevity and stress resistance of WT *S. pombe* in CR or in mutants of the glucose-signaling pathway. Longer chronological life span correlates with higher mitochondrial respiration and increased oxygen stress resistance, except with Gpa^R176H^ strain where loss of survival correlates only with weaker oxidative stress resistance. (A) CLS of *Δgit3* grown in 2% and 0.2% glucose as compared to wild type. (B) Chronological life span of *gpa2^R176H^* grown in 2% and 0.2% glucose. (C) Oxidative stress resistance measured in WT, *Δgit3* and *gpa2^R176H^* after short treatment of H_2_O_2_ and menadione. Cells were collected at day 1 of stationary phase and submitted to oxidative stressors before plating. (D) Oxidative stress resistance measured in WT and *gpa2^R176H^* at weak dose of H_2_O_2_ and menadione. (E) Transcript levels of cytosolic and mitochondrial superoxide dismutase (SOD1 and 2) normalized to wild type cells as measured by quantitative RT-PCR. Total RNAs were isolated in cells collected at day one of stationary phase. The transcript levels were normalized on two constitutively expressed genes. Standard deviations were calculated on three independent experiments; student test was done comparing with wild type at glucose 2%. *, *p*<0.01.

Guarente and colleagues proposed that in yeast, CR increases life span by increasing respiration but not oxidative stress resistance [17]. However, another study from Kaeberlein and collaborators contradicted these data. They showed that reducing glucose levels increased replicative life span in respiratory-deficient yeast [47]. We showed above that both CR and the *Δgit3* mutation increase respiration, while the *gpa2* ^R176H^ mutation decreased the effect of CR on respiration. Combining CR and the *Δgit3* mutation did not increase respiration over the values with CR alone. However, the survival of *Δgit3* cells was higher on CR than that of WT cells (Figure 4A). On the other hand, CR did not increase the respiration rate of the strain expressing activated Gpa2^R176H^p as this intervention did in WT cells (Figure 3D), and this defect could partially explain its short life span (Figure 4B).

To investigate further whether the additive effect of CR and loss of Git3p signaling involves respiration, cells were cultured in 20 µM of antimycine A, an inhibitor of complex III of the mitochondrial electron transport chain, that creates a leakage of electrons [48] and increases ROS production. Glucose restriction and *git3* ^+^ deletion together increased longevity in this high-ROS context (Figure S5). This suggests that low glucose signaling cooperates with other effects of CR acting downstream of ROS production, perhaps stimulating ROS defense mechanisms. Together, the data suggests that CR and reduced glucose signaling are not equivalent and these manipulations can actually cooperate to increase life span by a mechanism different than an increase in respiration.

To investigate whether resistance to oxidative stress could explain the longevity effects of CR and *git3* ^+^ deletion, we next study the effects of several pro-oxidants molecules on WT and mutants *S. pombe* cells grown at high or low glucose concentrations. First, CR and, to a lesser extent, loss of the Git3p GPCR increased hydrogen peroxide and menadione resistance (Figure 4C). Moreover, CR strengthened the already high stress resistance of *Δgit3* cells (Figure 4C). On the other hand, the resistance to both hydrogen peroxide and menadione treatment in *gpa2* ^R176H^ cells was significantly lower than in WT (Figure 4D). This stress sensitivity could also explain the very short CLS of this mutant in both high and low glucose.

We also measured the levels of cytosolic Cu/Zn-superoxide dismutase (SOD1) and mitochondrial Mn-SOD (SOD2) by quantitative PCR (Figure 4E). The importance of these two enzymes for long-term survival was demonstrated in budding yeast cultured in high glucose concentration [49]. No significant differences of expression were seen, neither in SOD2 (Figure 4E) nor in glutathione peroxidase (Gpx1, *data not shown*) for all the mutants and growth conditions tested. Interestingly, WT cells on CR showed no increased expression of SOD1 or SOD2 despite their very high oxidative stress resistance (Figure 2C). On the other hand, the deletion of glucose receptor increased significantly SOD1 expression. This correlates with the gain of oxidative stress resistance of this strain (Figure 4C). An unexpected three to six time rise of SOD1 transcript was observed in the *gpa2^R176H^* mutant, even if this strain displayed a very weak oxidative stress resistance. This could be the consequence of a feedback mechanism attributable to the very high production of ROS in this strain (Figure 2C). Although further studies on the mechanisms of stress resistance will be necessary, our data clearly shows that glucose signaling regulates SOD1 expression in *S. pombe*. Since SOD1 is not regulated by CR, it may be part of the mechanism by which the *git3* ^+^ deletion cooperates with CR to increase the resistance to oxidative stress and life span.

### Loss of Hexokinase 2 Activity Decreases Glucose Metabolism, Increases Glucose Signaling and Promotes Aging

**Figure 5:**
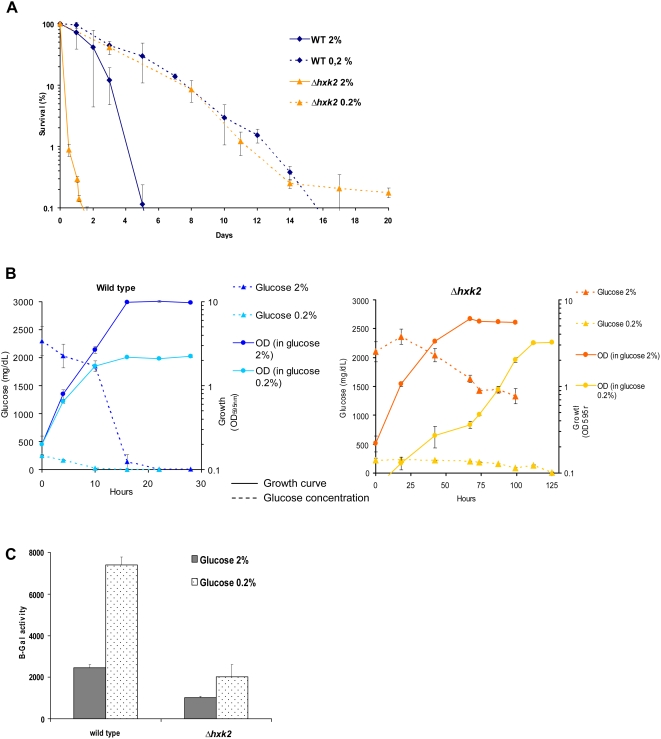
Hexokinase 2 mutant has a decreased survival and an increased glucose signaling in stationary phase. Impairing glucose metabolism strongly decreases CLS and increases Git3/PKA signaling in late exponential phase cells. This pro-aging effect is lost when less glucose is provided. (A) CLS of WT and *Δhxk2* in low and high glucose concentration. (B) Glucose consumption according to growth in WT and *Δhxk2* cultured in 2% and 0.2% glucose. (C) β-Galactosidase activity of *Δhxk2* with *fbp1-lacZ* reporter. Cells were collected at late exponential phase and β-galactosidase activity was measured.

In yeast, hexokinase 2 is responsible for channeling glucose into metabolic pathways by catalyzing phosphorylation of this sugar. It also has a function in glucose signaling in *S. cerevisiae* by promoting the down-regulation of glucose-repressed genes [50],[51]. Mutants of hexokinase do not influence CLS but increase replicative life span in *S. cerevisiae* [17],[43]. In fission yeast, the glucose phosphorylation activity is provided by two hexokinases (Hxk1p and Hxk2p), but the main enzymatic activity is due to hexokinase 2 [50]. Loss of Hxk1p has no significant phenotype (*data not shown*) and loss of Hxk2p dramatically decreases the growth rate in glucose [50]. The double knockout of both *hxk1* ^+^ and *hxk2^+^* is not viable on glucose [50]. To determine if hexokinase affects CLS in *S. pombe*, we first measured the life span of an *S. pombe Δhxk2* deletion strain. Unlike in *S. cerevisiae*, we observed a significant decrease in CLS in this strain (Figure 5A).

We have shown above that glucose signaling mediates pro-aging effects in *S. pombe*. Therefore, we reasoned that defective glucose utilization in the Δ*hxk2* strain could result in an accumulation of intracellular glucose followed by the inhibition of glucose import. Moreover in *S. cerevisiae*, glucose can be re-exported in the extracellular medium by the *hxt* hexose transporter [52]. In turn, the high extracellular glucose concentration would lead to increase the duration of glucose signaling. To test this hypothesis, we first measured the glucose concentration in the medium during the growth of both wild-type and *Δhxk2* cells. We found that glucose levels remained high in the *Δhxk2* culture as compared to WT, and that this strain has a very slow growth rate (Figure 5B). Congruently with this observation, at early stationary phase *Δhxk2* cells exhibited increased glucose signaling in comparison with control cells, as represented by the drop of *fbp1-lacZ* reporter expression (Figure 5C). In budding yeast, hexokinase activity has been involved in glucose-signaling pathways during exponential phase [53]. Our results do not contradict, but support those observations since the *Δhxk2* mutant has a defect in glucose signaling in exponential phase when compared to wild type, as indicated by elevated *fbp1-lacZ* expression (*data not shown*). However, the *Δhxk2* mutant reaches stationary phase with glucose in the medium and its short life span was completely rescued when cultured in 0.2% glucose. As expected, culturing *Δhxk2* cells in low glucose resulted in a two-fold increase in β-galactosidase activity indicating an increase in *fbp1-lacZ* reporter expression. This shows a reduction in signaling through the Git3/PKA pathway (Figure 5A and 5C). Taken together, the results are consistent with the model that an increase in glucose signaling via the Git3/PKA pathway accelerates aging in *Δhxk2* mutants.

### Glucose GPCR Git3p Promotes Aging in Absence of Glucose Metabolism

The strain *Δhxk2* still has the hexokinase 1 activity permitting glucose metabolism (Figure 5B) [50]. To confirm the importance of the pro-aging effect of glucose signaling isolated from the effect of glucose utilization as energy source, we constructed a double knockout of both hexokinases in fission yeast (*hxk1^+^* and *hxk2^+^*). These two mutations should prevent glucose from entering glycolysis and the pentose phosphate pathway. However, so far attempts to obtain this double mutant has been unsuccessful [50]. It was concluded that *Δhxk1 Δhxk2* strain is not viable on glucose. To circumvent this problem, we complemented *Δhxk2* with a plasmid expressing Hxk2p (pREP41_*hxk2* ^+^) and crossed it with a *Δhxk1* strain. After sporulation of the diploid, we selected for offspring containing both *Δhxk1* and *Δhxk2* deletions and the plasmid pREP41_ *hxk2* ^+^. Then we allowed the strain to lose the *hxk2^+^* plasmid in a medium containing only glycerol as carbon source and picked clones without plasmid. Because we obtained viable double mutants, we concluded that hexokinase activity and possibly glucose phosphorylation was required for sporulation but not for survival in *S. pombe*. Using the same approach, the triple knockout, *Δhxk1 Δhxk2 Δgit3* was created.

These mutants *Δhxk1 Δhxk2* and *Δhxk1 Δhxk2 Δgit3* could not grow when switched on plates with only glucose as carbon source. After at least ten days of incubation however, in some plates we observed for both strains clones that grew on glucose at a frequency between 10^−6^ to 10^−7^ (*data not shown*). The appearance of such clones was attributed to genetic reversion due to the nature of hexokinase 2 knockout that was created by insertion of a marker rather than complete suppression of the open reading frame [50].

**Figure 6:**
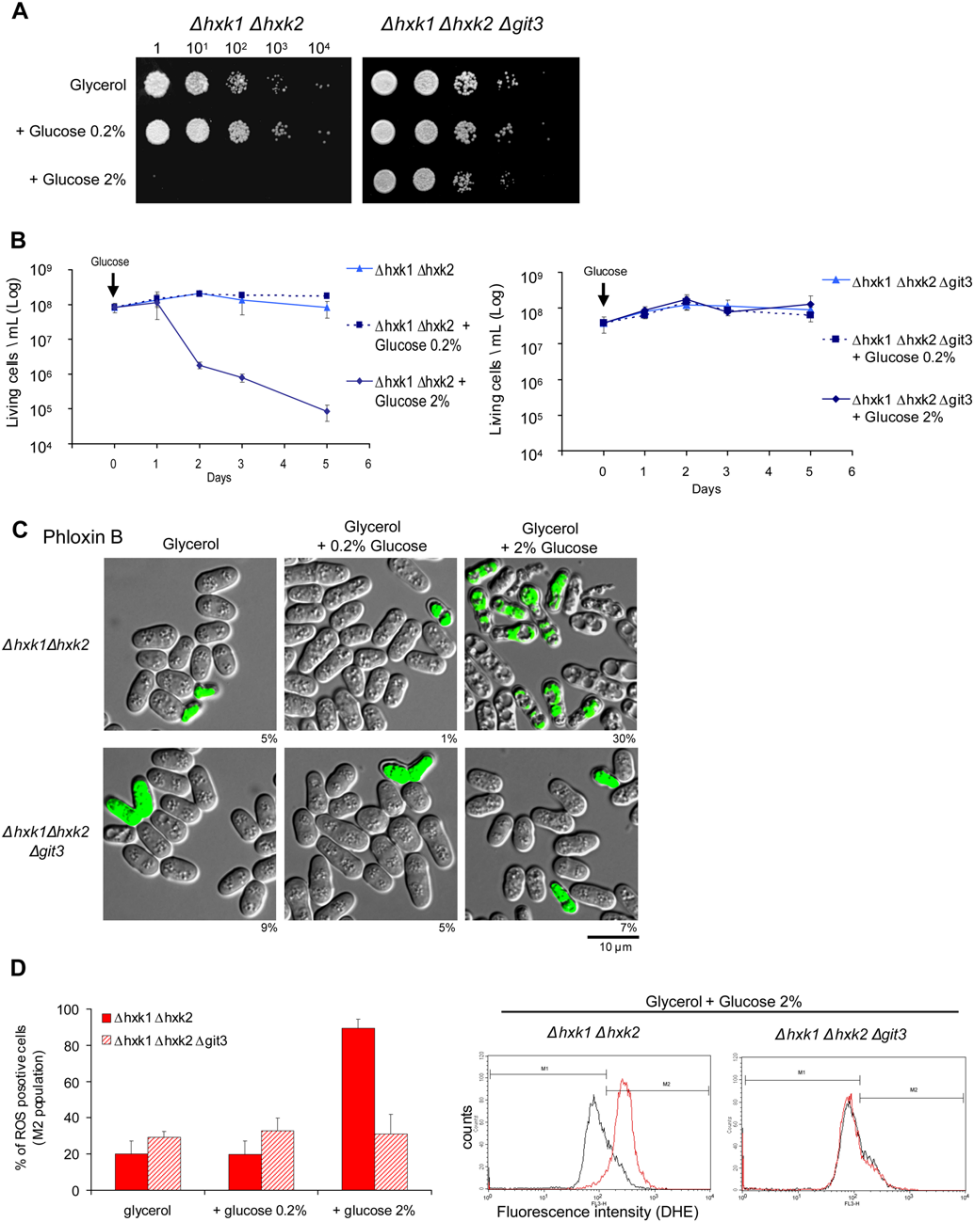
Git3p-dependent glucose signaling promotes aging in the absence of glucose metabolism. (A) Survival of *Δhxk1 Δhxk2* and *Δhxk1 Δhxk2 Δgit3* cells shifted from liquid culture with glycerol to solid media with glycerol, without or with glucose 0.2% or 2%. The growth of double hexokinase mutant on respiratory media plus glucose 2% is possible only with deleted Git3p-mediated signaling. 1 to 10^4^ represents the factors of dilution. (B) Quantification of living cells of *Δhxk1 Δhxk2* and *Δhxk1 Δhxk2 Δgit3* grown in respiratory media supplemented with glucose. The number of living cells per mL is calculated as the number of colony forming units on solid glycerol medium per mL of culture. Averages and standard deviations were calculated on three independent cultures. Left panel, *Δhxk1 Δhxk2*; right panel, *Δhxk1 Δhxk2 Δgit3*. (C) Microscopy analysis of double hexokinase mutants with vital dye Phloxin B applied 18 hours after glucose addition. The percentage under each picture represents the ratio of fluorescent cells counted under microscope on 500 cellules. (D) ROS production estimated by flow cytometry using DHE staining. This assay was carried out 36 hours after glucose addition. Left panel, increase of DHE staining was observed only in Δ*hxk1 Δhxk2* with glucose 2%, corresponding to premature aged cells. Right panel, summary of flow cytometry results of DHE-fluorescing cells (M2 population). In Δ*hxk1 Δhxk2*, 90% of the cells displayed about a 3 times greater fluorescence intensity between no glucose and glucose 2%. Three independent cultures were analysed for averages and standard deviations.

Mutants *Δhxk1 Δhxk2* and *Δhxk1 Δhxk2 Δgit3* were grown in glycerol as a carbon source for around two divisions with a doubling time of around ten hours. At this point, they were spotted on plates containing glycerol plus glucose (Figure 6A). The double mutant *Δhxk1 Δhxk2* did not grow on glycerol with 2% glucose, but it did on glycerol with 0.2% glucose. This result is consistent with the idea that the absence of hexokinase activity leads to a sustained and toxic glucose signaling. In agreement, the impaired growth of the double hexokinase mutant on glycerol plus glucose 2% was completely restored by a deletion in the glucose receptor *git3^+^* (Figure 6A).

To assess whether the increase in glucose signaling of the double hexokinase knockout decreases the viability in stationary phase (chronological aging), we added 2% glucose to liquid cultures at late exponential phase (OD_595_ 5–6). Then, viability was evaluated as a function of time by counting the number of living cells per mL (Figure 6B). After glucose addition, cultures with and without glucose needed two-day incubation to reach saturation corresponding to OD_595_ 13 to 16. The *Δhxk1 Δhxk2* double deletion mutant exposed to 2% glucose displayed striking loss of viability 24 hours after glucose addition in comparison to cultures with no added glucose (Figure 6B). This loss of viability was prevented by CR (0.2% glucose) or by deletion of *git3^+^* (Figure 6B). To further characterize the loss of viability induced by glucose in double hexokinase knockout strains, we stained yeast cells with Phloxin B, a dye accumulated by dead cells. We found a high proportion of stained cells (30%) at 18 hours after glucose addition in comparison to 5% in control cells (Figure 6C). Notably, Phloxin B stained cells were longer and displayed oversized vacuoles, a typical phenotype of aging in yeast (Figure 6C). ROS production was evaluated 36 hours after glucose addition by flow cytometry with DHE staining. A considerable number of DHE stained *Δhxk1 Δhxk2* cells was observed in the culture with 2% glucose (Figure 6C). Again, Phloxin B staining and the increase in ROS were prevented by CR (glucose 0.2%) or deletion of *git3^+^* (Figures 6B and C).

Our results show that glucose signaling via the Git3p GPCR is required for the pro-aging effects of glucose in *S. pombe* and is sufficient to mediate detrimental effects even in the absence of glucose consumption.

## Discussion

Excessive glucose signaling has been associated with humans diseases such as diabetes, as well as with the less understood process of aging [54]. Several mechanisms have been proposed for the harmful effects of glucose. Glucose can be directly toxic to cell components because it can promote non-enzymatic glycosylation and the accumulation of advanced glycation end products (AGE) which impair cellular functions [55],[56]. Excess glucose metabolism can also be deleterious because glucose oxidation increases the source of electrons to the mitochondrial respiratory chain in the form of NADH. In cells with a very active glucose metabolism, excess electrons can promote the generation of deleterious ROS if there is no matching increase in the efficiency of electron transport [54],[57]. Glucose and/or nutrient-signaling pathways also control life span in various species including yeast [15]. The data raise the question about the relative contribution of signaling and metabolism to the regulation of life span [58].

**Figure 7:**
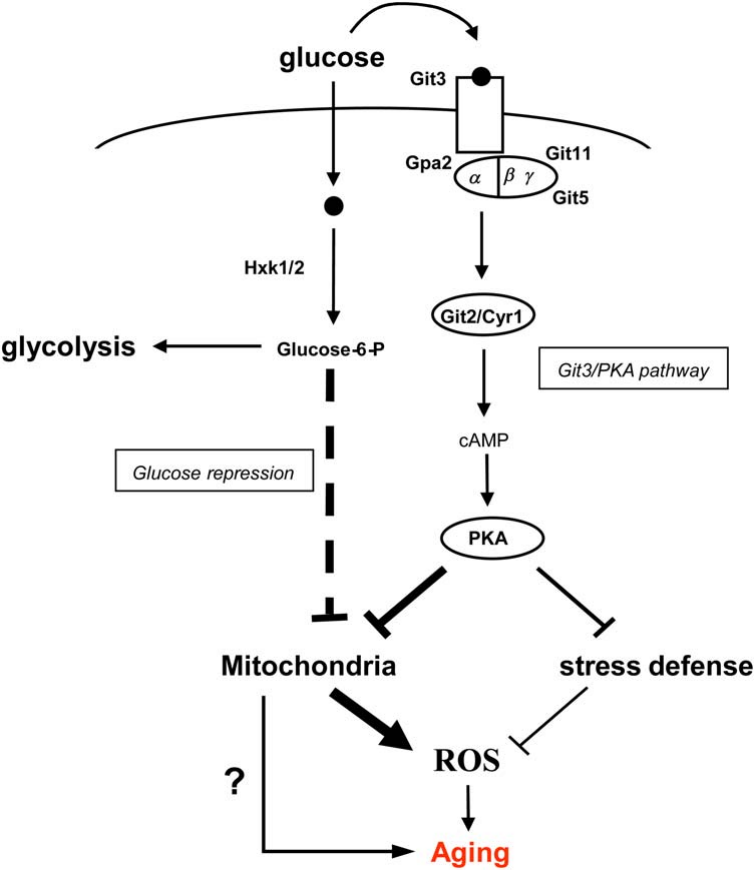
A glucose-signaling pathway involved in fission yeast longevity. Glucose availability is sensed through two major pathways in fission yeast, the Git3/PKA pathway and the glucose repression pathway. Each of these pathways regulates mitochondrial functions. In the present study, we underlined the importance of Git3/PKA dependent signaling in longevity versus the glucose repression pathway and the metabolic effect of glycolysis. Git3/PKA signal inhibits mitochondrial respiration, oxidative stress response and promotes ROS formation and pro-aging phenotypes. Discontinuous line represents functions inferred by homology with *S. cerevisiae*. Question mark represents the possible role of mitochondria in aging besides ROS production.

Here we examined this question in *S. pombe* using mutants of the Git3/PKA glucose-signaling pathway. In this pathway, PKA kinase is activated by glucose signaling through the Git3p G protein-coupled receptor (GPCR), which results in the Gα subunit (Gpa2p)-mediated activation of adenylate cyclase [29] as represented in Figure 7. This, in turn, produces a linear increase in cAMP levels. The cAMP is bound by the Cgs1 regulatory subunit of Pka1 kinase, activating PKA. The consequence is a re-localization of PKA to the nucleus followed by the inhibition of the Rst2 transcription factor, an increase in stress sensitivity and a decrease in cell survival [21],[33],[59]. We previously demonstrated the importance of cAMP/PKA pathway in regulating *S. pombe* aging by showing that knocking out the only catalytic subunit of the PKA complex results in increased chronological life span as well as enhanced stress resistance [21]. However, other nutrient-signaling pathways may activate PKA complex in yeast, so the specific role of glucose signaling in the longevity of *S. pombe* was unknown.

We show here that low glucose levels increase CLS in *S. pombe*, a typical CR response. Also, mutants with a defective Git3/PKA pathway have an increased life span, a normal glucose consumption rate, and only a slightly reduced growth rate. The reverse is also true. High glucose concentration, acting through the Git3/PKA pathway, promotes aging and decreases stress defense and respiration. Likewise a constitutively active Gα subunit, normally coupled to the Git3p GPCR, mimics the effects of high glucose even in low glucose.

Further support for a role of glucose signaling in the control of CLS in *S. pombe* was obtained by studying hexokinase deletion strains. These mutants die prematurely in stationary phase concomitant with prolonged stimulation of Git3/PKA signaling. Since cells without hexokinase cannot metabolize glucose, these results suggest that sustained glucose signaling, caused by the excess of extracellular glucose that remains in the medium of hexokinase mutants, promotes aging in *S. pombe*. The loss of Git3p GPCR blocks the detrimental effects of glucose in double hexokinase mutant. This suggests that glucose exerts a strong pro-aging effect via the Git3/PKA signaling pathway. Notably, the premature death of double hexokinase mutant due to high glucose is concomitant with an accumulation of ROS.

It is remarkable that the effect of deleting hexokinases differs between *S. pombe* and *S. cerevisiae*. In the budding yeast, deletion of all major hexokinases (glucokinase, hexokinase 1 and 2) impairs cAMP production and activation of the PKA pathway [60]. Conflicting with these data, we show that in *S. pombe*, hexokinase mutants die prematurely due to sustained signaling through this pathway. Careful examination of our results also reveals that hexokinase mutants have a defective PKA pathway during the exponential phase of the cultures. However, these mutants in *S. pombe* enter stationary phase with high concentrations of glucose in the medium and a continual activity of the Git3/PKA pathway that is responsible for their premature aging. In contrast, hexokinase mutants in *S. cerevisiae* have longer RLS and a normal CLS [8],[43]. This apparent discrepancy could be the result of particular differences in the glucose-signaling pathways and energy metabolism between *S. pombe* and *S. cerevisiae*. For instance, the regulation of glycolysis is different between these two yeasts. *S. cerevisiae* growth on glucose is sensitive to trehalose biosynthesis whereas *S. pombe* is not [61].

Despite the very significant role of Git3/PKA pathway in the pro-aging effect observed in the double hexokinase mutant, our work showed that the signal from the Git3p GPCR dependent pathway is not the only regulator of all the effects on aging due to glucose. First, in minimal medium completed (SDC), lowering glucose concentration had no effect on longevity (*data not shown*). Nevertheless, glucose decreased longevity when *S. pombe* were grown in synthetic medium based on yeast nitrogen base [26]. Other nutrient limitation is suspected to affect PKA-regulated processes. For instance, conjugation efficiency is controlled in both Git3/PKA cAMP-dependent manner and in a cAMP/PKA independent manner sensitive to medium composition [62]. This PKA-independent nutrient sensing could mimic the effect of glucose restriction in rich medium and may explain why the life span of *S. pombe* grown in SDC is not affected by glucose. This explanation is consistent with the fact that the respiration rate in 2% glucose is higher in synthetic medium than in rich medium (complex medium) [26]. Another indication that the signal from the Git3/PKA pathway is not the only one to control the rate of aging is provided by the glucose receptor mutant (*Δgit3*). This deletion strain still responds to CR with a higher oxidative stress resistance, lower ROS levels and an increased survival (Figure 4C, 2C and 4A). In agreement, studies in *Caenorhabditis elegans* [63], *Drosophila* [64] and mice show that disabling the insulin/IGF-1 signaling pathway can cooperate with CR to increase longevity [65]–[72]. What are the other possible mechanisms by which glucose could accelerate aging in *S. pombe*?

In budding yeast, glucose activates the glucose repression pathway, which is regulated by the AMP-activated protein kinase (AMPK) Snf1p complex [53],[73]. So the Git3/PKA-independent effect of glucose could be explained by the activation of AMPK complex which affects aging in yeast and metazoans [74],[75]. Our *S. pombe* hexokinase deletion mutants are expected to be defective in this pathway [53],[76] but they still age prematurely when grown in high glucose concentrations, suggesting that glucose repression is not involved in the pro-aging effects of glucose. Conversely, we could not discard the possibility that some pro-aging effects of glucose are mediated by the non-enzymatic glycosylation of proteins by glucose. Nevertheless, altogether our data point toward a regulation of longevity primarily via the glucose signaling through Git3/PKA pathway, raising the question about the underlying mechanisms. Although further work is required to discover the mechanisms by which glucose signaling accelerates aging in *S. pombe*, our current evidence points to the mitochondria as the target of glucose signals. First, CR (low glucose) in wild type yeast enhances respiration and mitochondrial membrane potential, prevents ROS production and improves oxidative stress defense. Second, *Δgit3* cells have a similar phenotype and, in addition this strain displays a higher expression of cytosolic superoxide dismutase in stationary phase. These could explain the additional longevity of *Δgit3* cultured under CR conditions (Figure 7).

In yeast, the cAMP/PKA glucose sensing pathway possibly represents the ancestor pathway of insulin/IGF-1 signaling in multicellular eukaryotes. This pathway signals the presence of glucose, the preferred energy source. It also controls stress resistance, growth rate and sexual development, modifies mitochondrial metabolism, and ultimately controls life span as we have shown in this study. Similar to our observations for the Git3/PKA pathway in fission yeast, a decrease in the insulin/IGF-1 signal increases longevity as a function of CR in mammals. The extent to which dietary restriction may actually be effective in humans is still unknown. Our results also show that CR and loss of the Git3p GPCR cooperate to increase life span. This suggests that if this pathway is conserved in higher organisms, its inhibition may lead to an anti-aging treatment not relying on strict diets with a limited caloric content as used in animal research. Interestingly, inhibition of cAMP synthesis by the knockout of the type 5 adenylyl cyclase (AC5) gene induced Raf/MEK/ERK-dependent stress resistance and lengthened life span in mice [77]. The effect of reducing glucose signaling in *S. pombe* also results in a decreased cAMP synthesis in response to glucose, because Git3p, via Gpa2αp, activates adenylate cyclase [32]. Since CR and inhibition of glucose signaling cooperate to extend life span in *S. pombe*, it would be interesting to combine agents that reduce cAMP synthesis or reduce PKA activity with CR in mammals.

In conclusion, our work with *S. pombe* highlights the importance of glucose-signaling pathways and oxidative stress resistance in aging. Given the importance of glucose as a central metabolite, it is surprising that the pathway for glucose sensing existing in *S. pombe* has not been found yet in mammals. Whether a glucose receptor contributes to these signaling pathways in metazoans remains to be demonstrated. Our data together with the interesting phenotype of the AC5 KO mice provide the rationale for further inquiry into glucose sensing pathways in mammals.

## Materials and Methods

### Ethics Statement

This study was conducted according to the principles expressed in the Declaration of Helsinki.

### Media and Yeast Strains

MM refers to Edinburgh Minimal Medium [78] complemented by adenine, uracil, leucine and/or histidine 75 mg.L^−1^ (A,U,L,H). SMC refers to synthetic medium complemented and is composed of MM plus adenine, uracil, leucine and/or histidine 444 mg L^−1^ (A,U,L,H). Its composition is described in a previous study [21]. The same medium with glycerol 3% ethanol 0.2% for carbon source was named SMC glycerol. Mating and sporulation were carried out on MEA plates (bacto malt extract 3%, glucose 0.4%, pH 5.5, supplemented by adenine, histidine, uracil, leucine 225 mg.L^−1^ each). Yeast extract complete medium (YEC), was made of yeast extract 5 g.L^−1^ (BD, Difco) supplemented with 222 mg.L^−1^ of adenine, uracil, leucine and histidine, and glucose 2% unless otherwise specified. All cultures were incubated at 30°C in a rotating incubator shaker at 250 rpm (New Brunswick instrument).

Growth curves represent the average of three independent cultures. Morphological analysis of wild type cells in YEC glucose 0.05%, 0.2%, 0.5% or 2% was done in 10 mL cultures in 50 mL conic tubes with air-permeable cap grown overnight. Early log phase refers to OD_595_ 0.5.

The strains used in this work are all described in supplementary Table 1. Wild type refers to strain SP14000, except for Figure 2B, 5C, S4 in which it refers to FWP87. The *gpa2* ^R176H^ (RWP1) [32] *Δgit3* deletion (CHP984) [28], *Δhxk1* and *Δhxk2* deletions (CJM387, CJM389) [50] alleles were previously described.

The double *Δhxk1 Δhxk2* mutant was constructed as follows. *Δhxk2* (CJM389) was transformed with a plasmid bearing the *hxk2^+^* ORF, previously amplified by PCR and inserted into the *SalI* site of pREP41 (pREP41_Hxk2). PCR primes sequences will be provided upon request. The *Δhxk2* pREP41_Hxk2 strain (SP14405) was mated with *Δhxk1* (CJM387). Corresponding diploids were sporulated in MES media and spores *hxk1::ura4^+^ hxk2::his3^+^* harbouring the pREP41_Hxk2 plasmid were selected on MMA media. Haploids were grown to saturation in liquid SMC supplemented with adenine and leucine 222 mg.L^−1^ and with glycerol 3%, ethanol 0.2% as carbon sources. Then, they were diluted in the same fresh medium and cultured a second time to saturation in order to force cells to lose pREP41_Hxk2 plasmid. At this point, clones without plasmids were selected on plates SMC AL glycerol. The loss of pREP41_Hxk2 plasmid was validated by verifying that these clones *Δhxk1 Δhxk2* (SP14483 and SP14493) cannot grow without leucine, the marker on the pREP41 plasmid. In addition, these clones cannot grow on SMC AL glucose 2%. The triple mutant *Δhxk1 Δhxk2 Δgit3* was obtained by first constructing a *Δhxk1 Δgit3* double knockout (SP14373) after mating the single mutants *Δhxk1* (SP14313) and *Δgit3* (SP14105). The resulting *Δhxk1 Δgit3* strain was crossed with *Δhxk2* pREP41_Hxk2 (SP14405) and the haploid strain *Δhxk1 Δhxk2 Δgit3* without plasmid was isolated as described previously for *Δhxk1 Δhxk2*.

Three independent cultures of each double hexokinase mutant *Δhxk1 Δhxk2* and *Δhxk1 Δhxk2 Δgit3*, were started in 75 mL (250 mL flask) YEC glycerol and incubated 24 hours. At OD_595_ 0.6, 0.1 mL were harvested, washed in sterile water and serial diluted to be plated as drop test on solid YEC glycerol 3% ethanol 0.2% glucose 2% or 0.2% or no glucose. Plates were incubated 8 days at 30°C. The same 75 mL cultures were grew until OD_595_ 5 to 6 and split in 3 times 25 mL cultures, one let with glycerol only, one complemented with glucose 2%, one with glucose 0.2%. These 18 cultures were then studied as described below.

### Reversion of the Growth defect in Glucose of *Δhxk1 Δhxk2* Mutants

The frequency of cells able to recover the ability to grow on glucose in *Δhxk1 Δhxk2* mutants was measured by plating serial dilution of 100 µL of a saturated culture of SP14383 and SP14393 on SMC AL glycerol and on SMC AL glucose and by counting colonies forming units. The average of the ratios of six independent clones of SP14383 and SP14393 was 1.6×10^−6^. Because of the very low frequency of this event and the long time revertants take to grow and reach a significant number, we consider that these revertants did not influence our data.

### Chronological Life Span Assays and Survival

The protocol for CLS measurement by CFU counting has been described previously [21] except that the first estimation of the number of living cells was delayed. Cells that reached maximal density were harvested, serial diluted and plated 24 hours and 48 hours after the optical density was stable and maximal; the higher number of living cells from these two samples was considered as the beginning of CLS curve (i.e., survival 100%). Error bars represent standard deviation calculated from four cultures separated from a single initial culture at the end of exponential phase. Each assay was repeated at least three times. All CLS analysis were performed in YEC AULH 222 mg L^−1^ except in Figure 1E where the medium is SMC AUL 444 mg.L^−1^ glycerol 3%. For antimycine A treatment, cultures were started at OD_595_ 0.2 with 20 µg.mL^−1^ antimycine A (solubilized in ethanol 100%) and CLS was measured as described above, except that cells entered stationary phase at a lower OD.

Number of living cells per mL was calculated by plating dilutions of sample of the cultures as described above accepted that solid YEC glycerol was used. The concentration presented (living cells/mL) with standard deviation represents the average of three independent cultures. Survival analysis by Phloxin B staining was done according to a previous publication [21], with the exception that the percentage of stained cell was obtained by counting under microscope after background subtraction. At least 500 cells were counted for each condition.

### *In vivo* Staining of ROS by DHR 123 and DHE

Epifluorescence microscopy analyses were performed using an inverted Nikon Eclipse E800 microscope equipped with a Nikon_60 DIC H (1.4 NA) lens and a Photometrics CoolSNAP fx CCD camera. Images were acquired using a motion-picture camera CCD CoolSnapFX 12 (Photometrics, Tucson, AZ, USA) bit and analysed with UIC Metamorph software (Molecular Devices Corporation, Downington, PA, USA). The percent of ROS-positive cells was measured with dihydrorodhamine 123 (DHR 123, Sigma) following a previously described protocol [21]. The fluorescence of this dye is activated by peroxynitrite and peroxide in the presence of peroxidase [79]. A total of 500 to 700 cells per culture were counted to determine the percentage of positively stained cells and standard deviations were calculated using three independent experiments. Staining by dihydroethidium (DHE, Sigma) is more specific to superoxide production [80] and was achieved as followed. 1.4×10^7^ cells were collected and resuspended in 0.1 mL 1× PBS with DHE 50 µM and incubated 10 minutes at 30°C. The DHE solution was removed, cells were resuspended in 20 µL 1× PBS and deposited on a microscope slide with a thin layer of agarose 1%. Counting was done the same way than for DHR123 using a Cy3 filter. Flow cytometry analysis was performed following the protocol detailed in [21] excepted for cells grown in glycerol. They were sonicated 15 seconds with a Sonicator Dismembrator Fisher Scientific Model 100 set to 12 watts. FACS analysis was done using FL1 filter for DiOC_6_ dye and FL3 filter for DHE dye.

### Oxygen and Glucose Consumption Assays

Oxygen consumption was measured in cultures grown to cell concentrations between OD_595_ 0.8 and 1.5. Cells were cultured in YEC to a given OD, and then the culture was diluted in its own medium if OD_595_ was greater than 1.5, or concentrated in its own medium by centrifugation if OD_595_ was less than 0.8. The goal was to measure the respiration of cultures with similar concentrations and in the exact medium in which the samples were taken. 10 mL of culture, sometimes diluted or concentrated, was incubated with gentle agitation at 30°C and 5 mL was loaded in the measurement chamber at 30°C with agitation. The oxygen consumption was followed with a Clark electrode YSI model 53 oxygen monitor until all oxygen was consumed in the chamber. The calibration of the Clark electrode for the maximum oxygen concentration (100%) was done on the air. The consumption was linear, the measure was recorded with a tracer Linear1100 and the slope was calculated for each sample. The result corresponding to the rate of respiration was normalized on the OD_595_ of the cells in the chamber and expressed in %O_2_.min^−1^.OD^−1^.

Glucose concentration was measured on the supernatants of cultures at different ODs following the protocol given in Quantichrom™ Glucose Assay Kit from BioAssay Systems®. The results presented are the averages of three independent cultures.

### β-Galactosidase Assays

β-galactosidase activity, expressed from the *fbp1-lacZ* reporter, was determined as previously described [36] except that cultures were grown in YEC to late exponential phase (OD_595_ 9 in glucose 2%, and OD_595_ 2 in glucose 0.2%). CHP1229 was grown to only OD_595_ 5.5 corresponding to the end of its exponential phase.

### Oxidative Stress Survival and Re-Growth on Glycerol Plate

Cells were cultured in YEC glucose 2% or 0.2% to stationary phase, and harvested 24 hours thereafter. Cultures were diluted to OD_595_ 0.5 to 0.8 in water and submitted to various oxidative shocks at 30°C. These include 1 M H_2_O_2_ for 120 minutes; 0.75 M Menadione for 180 min; 0.5 M H_2_O_2_ for 30 min or 0.3 M Menadione for 90 minutes. Then, cells were washed twice with 1 mL water and serially diluted tenfold four times. Each dilution was spotted on YEC plates and incubated five days at 30°C. For re-growth on glycerol plates, cells were grown in YEC glucose 2% to OD_595_ 0.5 and washed twice in water. Cells were serially diluted as described above and spotted on SMC AULH glycerol 3%.

### Mitochondrial Membrane Potential Analysis by Flow Cytometry

Mitochondrial membrane potential (Δψm) was measured with DiOC6 (Molecular Probes). Yeast strains were grown over-night in 10 mL YEC using 50 mL tubes with half screw cap to allow gas exchange. Early exponential phase refers to cells harvested at OD_595_ from 0.7 to 1. Late exponential phase refers to cultures at OD_595_ 2.1 to 2.4 in glucose 0.2% and OD_595_ 4 to 5 for cultures in 2% glucose. Stationary phase refers to cultures let 24 hours in the incubators after saturation, corresponding to OD_595_ 2.4 to 2.7 in 0.2% glucose and OD_595_ 6 to 7 in 2% glucose. Then, 1.4×10^7^cells were collected, concentrated in 0.1 mL of their own medium and incubated in DiOC_6_ 0.175 µM 15 minutes at 30°C. Next, 50 µL of this volume was diluted in 0.95 mL of 1× PBS and flow cytometry analysis was carried out as described above.

### Real Time Quantitative PCR

Total RNA were reverse transcribed in a final volume of 100 µL using the High Capacity cDNA Reverse Transcription Kit with random primers (Applied Biosystems, Foster City, CA) as described by the manufacturer. Reverse transcribed samples were stored at −20°C. A reference RNA (Human reference total RNA, Stratagene, Ca) was also transcribed in cDNA. Gene expression level was determined using primer and probe sets provided upon request. PCR reactions for 384 well plate formats were performed using 2 µL of cDNA samples (50 ng), 5 µL of the Express qPCR SuperMix (Invitrogen), 2 µM of each primer and 1 µM of the probe in a total volume of 10 µl. The ABI PRISM® 7900HT Sequence Detection System (Applied Biosystems) was used to detect the amplification level and was programmed FAST with an initial step of 3 minutes at 95°C, followed by 45 cycles of 5 seconds at 95°C and 30 seconds at 60°C. All reactions were run in triplicate and the average values were used for quantification. The relative quantification of target genes was determined using the ▵▵CT method. Briefly, the Ct (threshold cycle) values of target genes were normalized independently to endogenous control genes (▵CT = Ct _target_−Ct _endogenous_) and compared with a calibrator (WT 2% glucose sample C): ▵▵CT = ▵Ct _Sample_−▵Ct _Calibrator_. Relative expression (RQ) was calculated using the Sequence Detection System (SDS) 2.2.2 software (Applied Biosystems) and the formula is RQ = 2^−▵▵CT^. All gene expression (RQ) represents the average of three RQ from three independent experiments. Standard deviations were calculated with these three RQ. Two endogenous control genes were used: *Top1* ^+^ and SPBC887.02; both selected to be highly and constitutively expressed during stationary phase. Similar results were obtained with both of them. Results showed were calculated with *Top1* ^+^.

### RNA Extraction

5 mL of stationary phase culture (day 1) was resuspended in 300 µL guanidinium isothiocyanate Solution (Guanidinium Isothiocyanate 4 M, Sodium Citrate 25 mM, pH 7.0, β-Mercaptoethanol 1 M) in 2 mL screw cap tubes. 0.3 mL of RNase-free beads was added and vortexed 4 times 30 seconds with Bead Beater. All the homogenate was transferred to a 2 mL Phase Lock Tube (PLG) (Qiagen). 26 µL Sodium Acetate 2 M (pH 4.0) was added to the sample, cap the PLG tube and mix briefly. 260 µL water-saturated phenol was added to the sample, cap the PLG tube, and mix thoroughly. 75 µL Chloroform: Isoamyl Alcohol (49∶1) was added to the sample in the same PLG 2 ml tube and mix thoroughly by repeated gentle inversion. Incubate on ice for 15 minutes, and centrifuge at 13,000 rpm for 5 minutes in a microcentrifuge. The aqueous phase was transferred to a new pre-spin PLG 2 ml tube, 250 µL Phenol-Chloroform-Isoamyl Alcohol (50∶49∶1) was added and mixed thoroughly by repeated gentle inversion and centrifuge. In the same PLG tube, 250 µL Phenol-Chloroform-Isoamyl Alcohol (50∶49∶1) was added, then mixed and centrifuged. The resultant aqueous phase was collected; an equal volume of 100% Isopropanol was added, and mixed by repeated inversion. The solution was centrifuged at 13 000 rpm for 20 min at 4°C. The resultant supernatant was discarded and the pellet was washed 4 times with 200 µL 80% ethanol, using 2 minutes centrifugation to re-pellet the sample if necessary. The final wash was discarded and the pellet dried at room temperature. Finally, the pellet was dissolved in 100 µL RNase-free water and stored at −70°C. RNA integrity was checked on 1.5% agarose gel electrophoresis with RNA loading buffer (Qiagen).

### Accession Numbers

The code in parenthesis refers to pombe genome project nomenclature *git3^+^* (SPCC1753.02c); *gpa2* ^+^/*git8* ^+^ (SPAC23H3.13c); *hxk1* ^+^ (SPAC24H6.04); *hxk2* ^+^ (SPAC4F8.07c); *fbp1* ^+^ (SPBC1198.14c); *sod1* ^+^ (SPAC821.10c); *sod2* ^+^ (SPAC1486.01); *gpx1* ^+^ (SPBC32F12.03c); *top1* ^+^ (SPBC1703.14c); *unnamed a chloride channel* (SPBC1703.14c)

## Supporting Information

Figure S1 Growth curves of wild type in different concentration of glucose. The cells were grown in YES AULH and ODs were calculated on the average of three independent cultures.
(0.9 MB TIF)

Figure S2 Dihydrorhodamine 123 (DHR 123) and dihydroethidium (DHE) *in vivo* staining of reactive oxygen species. This control experiment was carried out in order to verify that DHR 123 and DHE were consistently specific of yeast cells that produce high quantity of ROS, alive or recently dead. Cells killed by amphotericin B treatment are not stained showing that dead cells are not systematically positive. (A) Comparison between the quantification of ROS-positive cells by DHR 123 or DHE. Cultures were stained at day 2 and 5 of the stationary phase and positive cells were counted under microscope (see Materials and Methods). Data shown are mean±standard deviation of three independent samples assayed. *, *p*<0.01, Student test, 2% glucose versus 0.2% glucose. (B) DHR 123 and DHE did not stain WT cells killed with amphotericin B. Cells were grown to stationary phase, treated or not with 10 µg/mL of amphotericin B during 16 hours (h), incubated in water during 24 h and analysed by fluorescence microscopy. Percents indicate the amount of ROS-positive cells as compared to the total number of cells. (C) Survival of WT cells after treatment with amphotericin B. Cells were grown to stationary phase, treated with 10 µg.mL^−1^ of amphotericin B during 16 h, incubated in water during 24 h, serially-diluted (10^1^ to 10^4^) and spotted on YEC glucose 2% plates. Growth was monitored during 5 days at 30°C. (D) Morphological comparison of ROS-positive and ROS-negative WT cells stained by DHR 123 or DHE. White arrows indicate marked cells that appear dead, black arrows indicate marked cells that appear alive and stars indicate unmarked cells that appear dead.
(8.9 MB TIF))

Figure S3 Glucose consumption according to growth of *gpa2^R176H^* and *Δgit3* yeasts grown in 2% and 0.2% glucose.
(1.2 MB TIF)

Figure S4 Mitochondrial membrane potential (Δψm) analysis by Flow cytometry on cells stained with DiOC_6_. Exp: exponential; Stat: stationary. Black arrows show dead cells. See Materials and Methods for details. DiOC_6_ is known to stain mitochondria in fission yeast [81]. The intensity of DiOC_6_ fluorescence is increasing with mitochondrial membrane potential, as shown in *S. cerevisiae* [82].
(2.1 MB TIF)

Figure S5 Chronological life span of WT and *Δgit3* grown in 2% and 0.2% glucose with 20 µg.mL^−1^ antimycine A (AA). Vector corresponds to ethanol to a final concentration to 0.1%.
(0.6 MB TIF)

Figure S6 β-Galactosidase activity of *Δgit3* and *gpa2^R176H^* with *fbp1-lacZ* reporter both grown in 2% and 0.2% glucose. Cells were collected at late exponential phase and β-Galactosidase activity was measured.
(0.7 MB TIF)

Table S1 Strains used in this study presented with their genotypes and the laboratory where they were created. * refers to strains created for this study.
(0.03 Mb DOC)
