# Supplementary figures and images for: Pro-Aging Effects of Glucose Signaling through a G Protein-Coupled Glucose Receptor in Fission Yeast
Source: PLoS Genet. 2009 Mar 6;5(3):e1000408. doi: 10.1371/journal.pgen.1000408 (PMC2646135; doi:10.1371/journal.pgen.1000408)

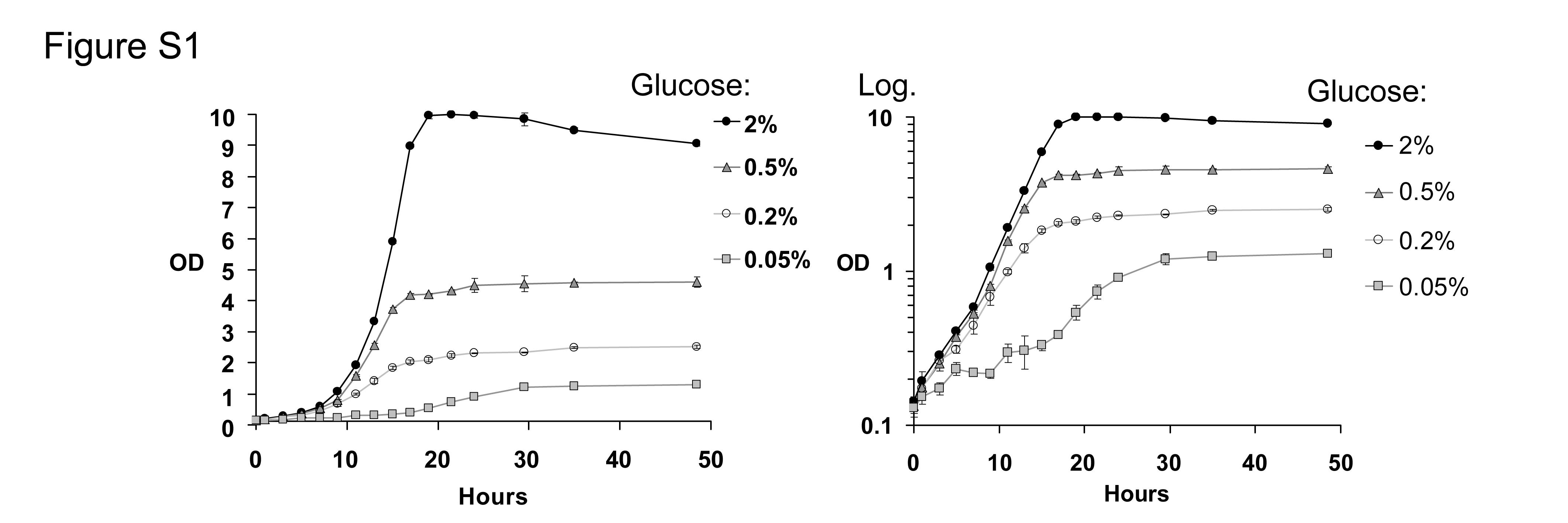

Supplement: Figure S1 — Growth curves of wild type in different concentration of glucose. The cells were grown in YES AULH and ODs were calculated on the average of three independent cultures. (0.9 MB TIF) [file pgen.1000408.s001.tif]

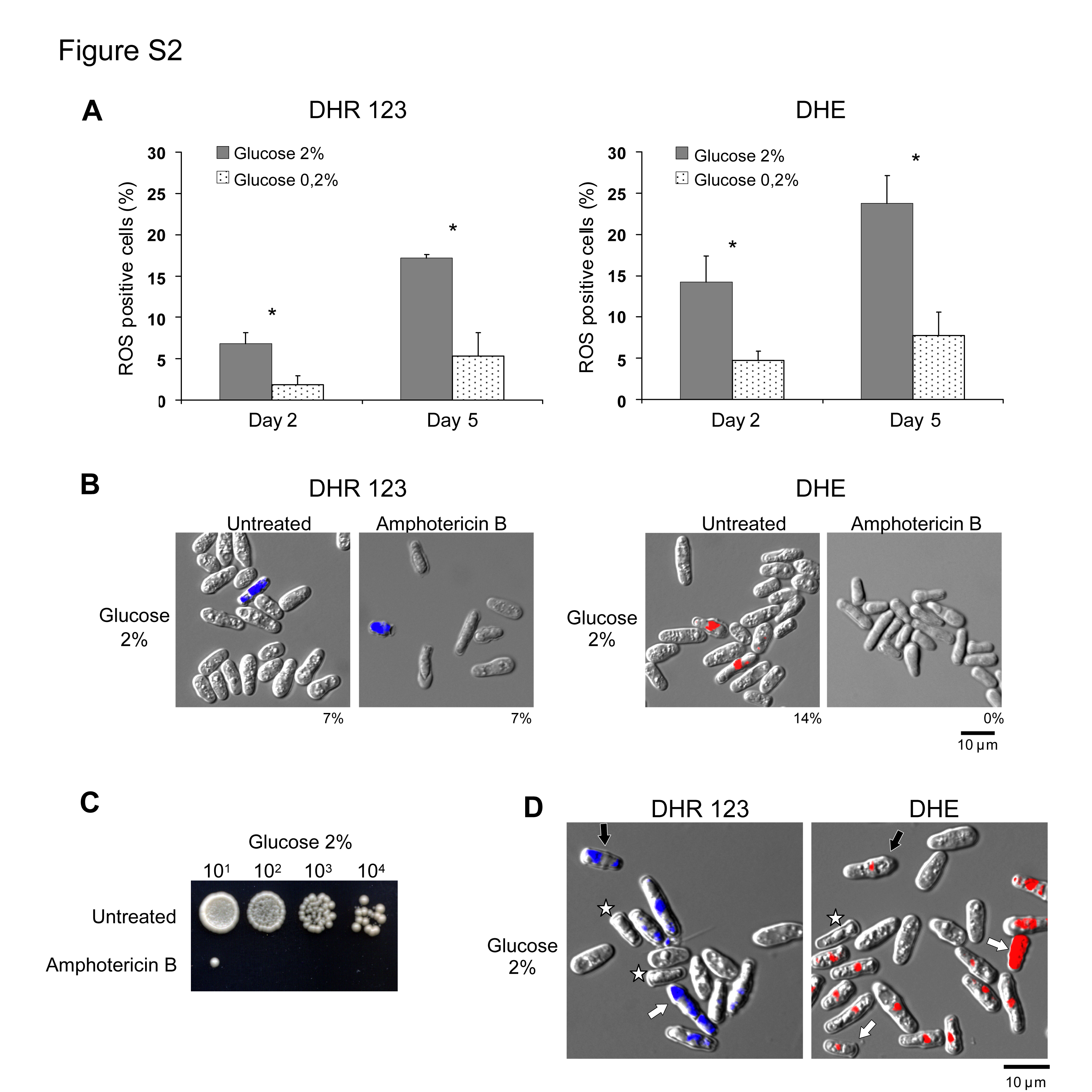

Supplement: Figure S2 — Dihydrorhodamine 123 (DHR 123) and dihydroethidium (DHE) in vivo staining of reactive oxygen species. This control experiment was carried out in order to verify that DHR 123 and DHE were consistently specific of yeast cells that produce high quantity of ROS, alive or recently dead. Cells killed by amphotericin B treatment are not stained showing that dead cells are not systematically positive. (A) Comparison between the quantification of ROS-positive cells by DHR 123 or DHE. Cultures were stained at day 2 and 5 of the stationary phase and positive cells were counted under microscope (see Materials and Methods). Data shown are mean±standard deviation of three independent samples assayed. *, p<0.01, Student test, 2% glucose versus 0.2% glucose. (B) DHR 123 and DHE did not stain WT cells killed with amphotericin B. Cells were grown to stationary phase, treated or not with 10 µg/mL of amphotericin B during 16 hours (h), incubated in water during 24 h and analysed by fluorescence microscopy. Percents indicate the amount of ROS-positive cells as compared to the total number of cells. (C) Survival of WT cells after treatment with amphotericin B. Cells were grown to stationary phase, treated with 10 µg.mL−1 of amphotericin B during 16 h, incubated in water during 24 h, serially-diluted (101 to 104) and spotted on YEC glucose 2% plates. Growth was monitored during 5 days at 30°C. (D) Morphological comparison of ROS-positive and ROS-negative WT cells stained by DHR 123 or DHE. White arrows indicate marked cells that appear dead, black arrows indicate marked cells that appear alive and stars indicate unmarked cells that appear dead. (8.9 MB TIF)) [file pgen.1000408.s002.tif]

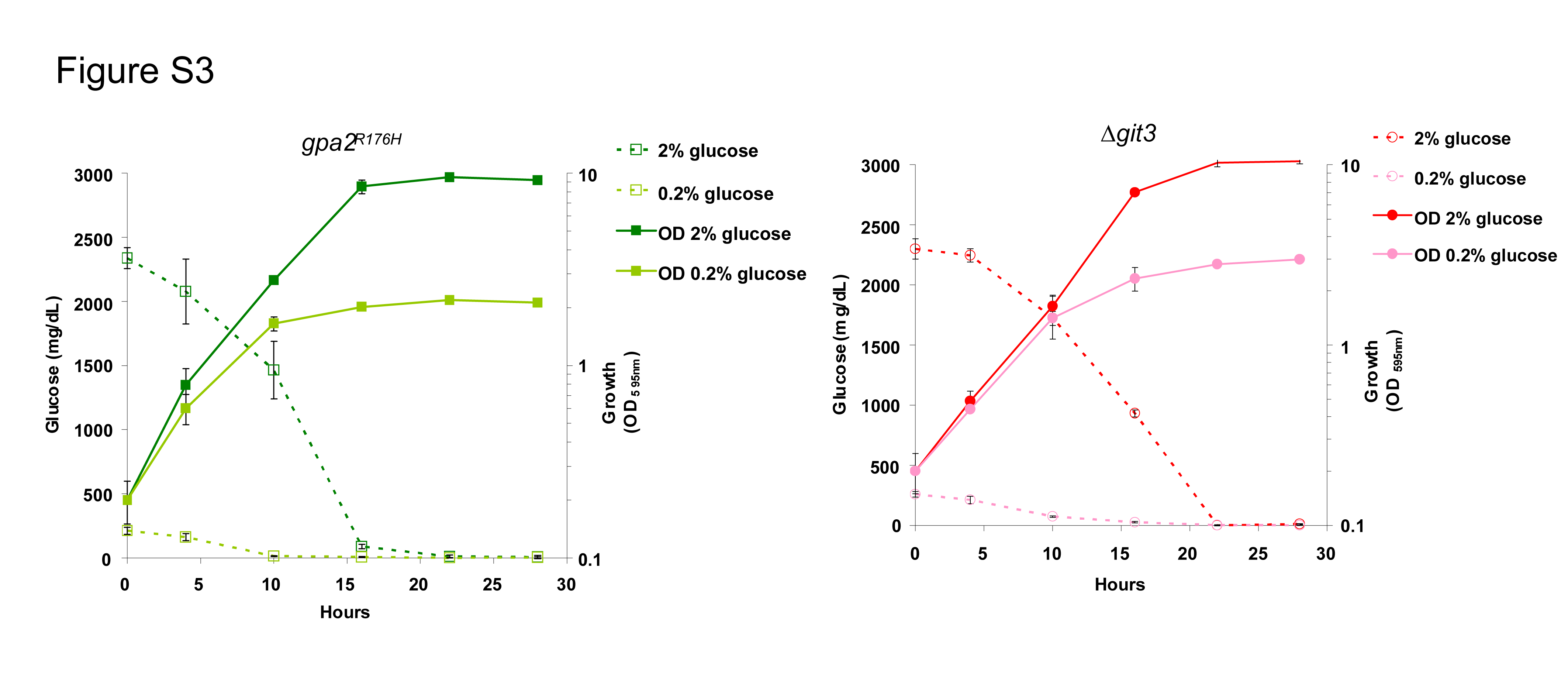

Supplement: Figure S3 — Glucose consumption according to growth of gpa2R176H and Δgit3 yeasts grown in 2% and 0.2% glucose. (1.2 MB TIF) [file pgen.1000408.s003.tif]

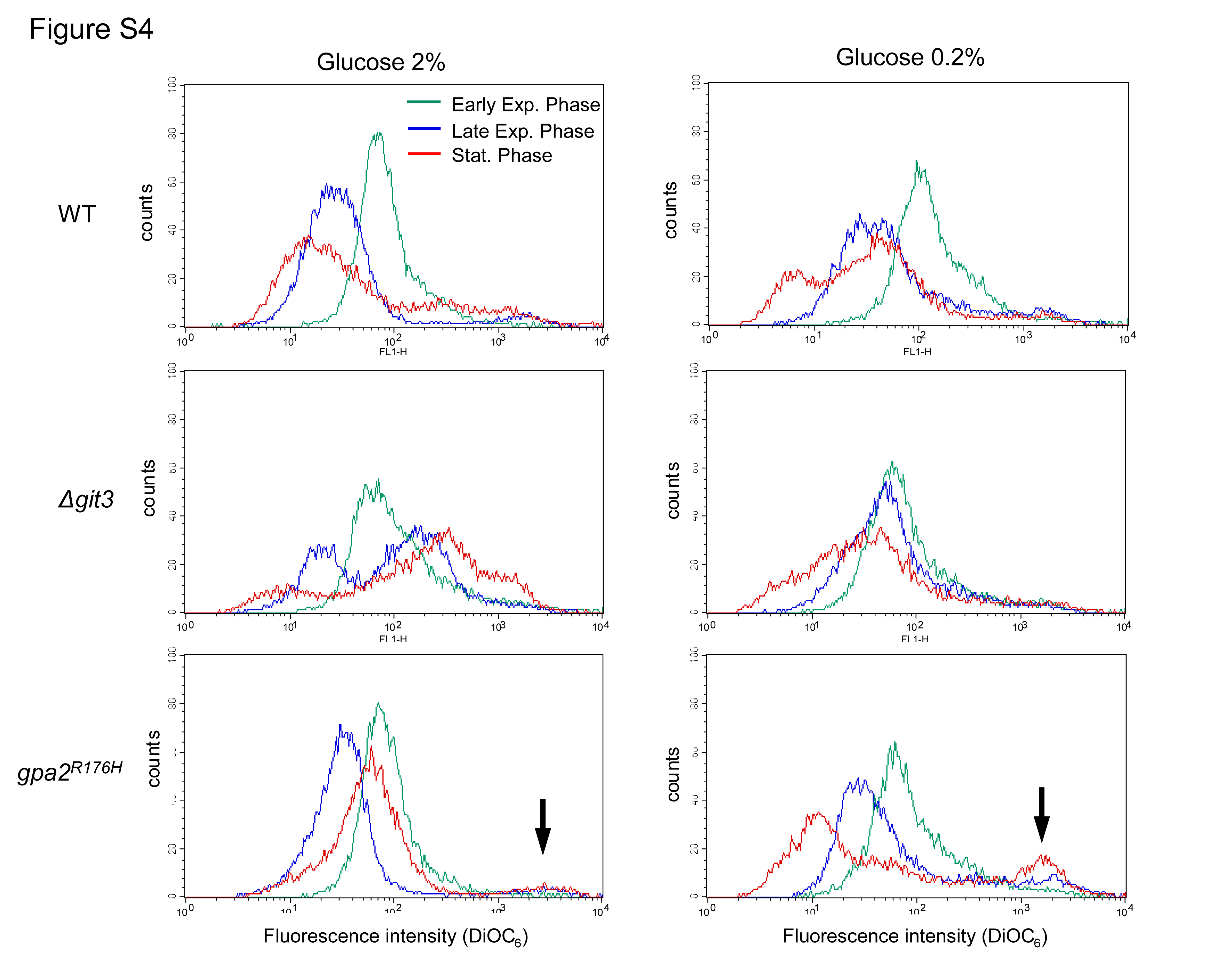

Supplement: Figure S4 — Mitochondrial membrane potential (Δψm) analysis by Flow cytometry on cells stained with DiOC6. Exp: exponential; Stat: stationary. Black arrows show dead cells. See Materials and Methods for details. DiOC6 is known to stain mitochondria in fission yeast [81]. The intensity of DiOC6 fluorescence is increasing with mitochondrial membrane potential, as shown in S. cerevisiae [82]. (2.1 MB TIF) [file pgen.1000408.s004.tif]

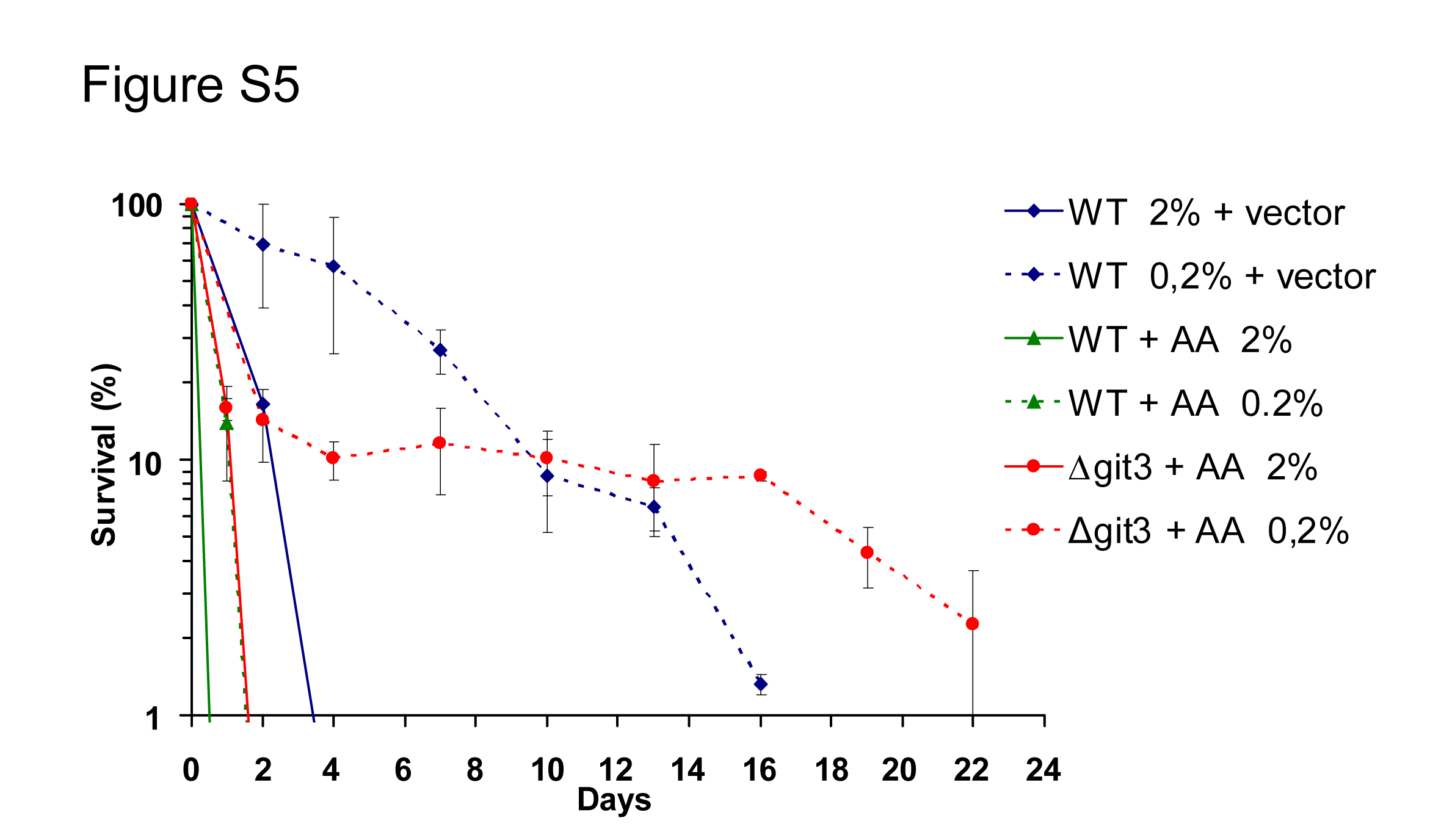

Supplement: Figure S5 — Chronological life span of WT and Δgit3 grown in 2% and 0.2% glucose with 20 µg.mL−1 antimycine A (AA). Vector corresponds to ethanol to a final concentration to 0.1%. (0.6 MB TIF) [file pgen.1000408.s005.tif]

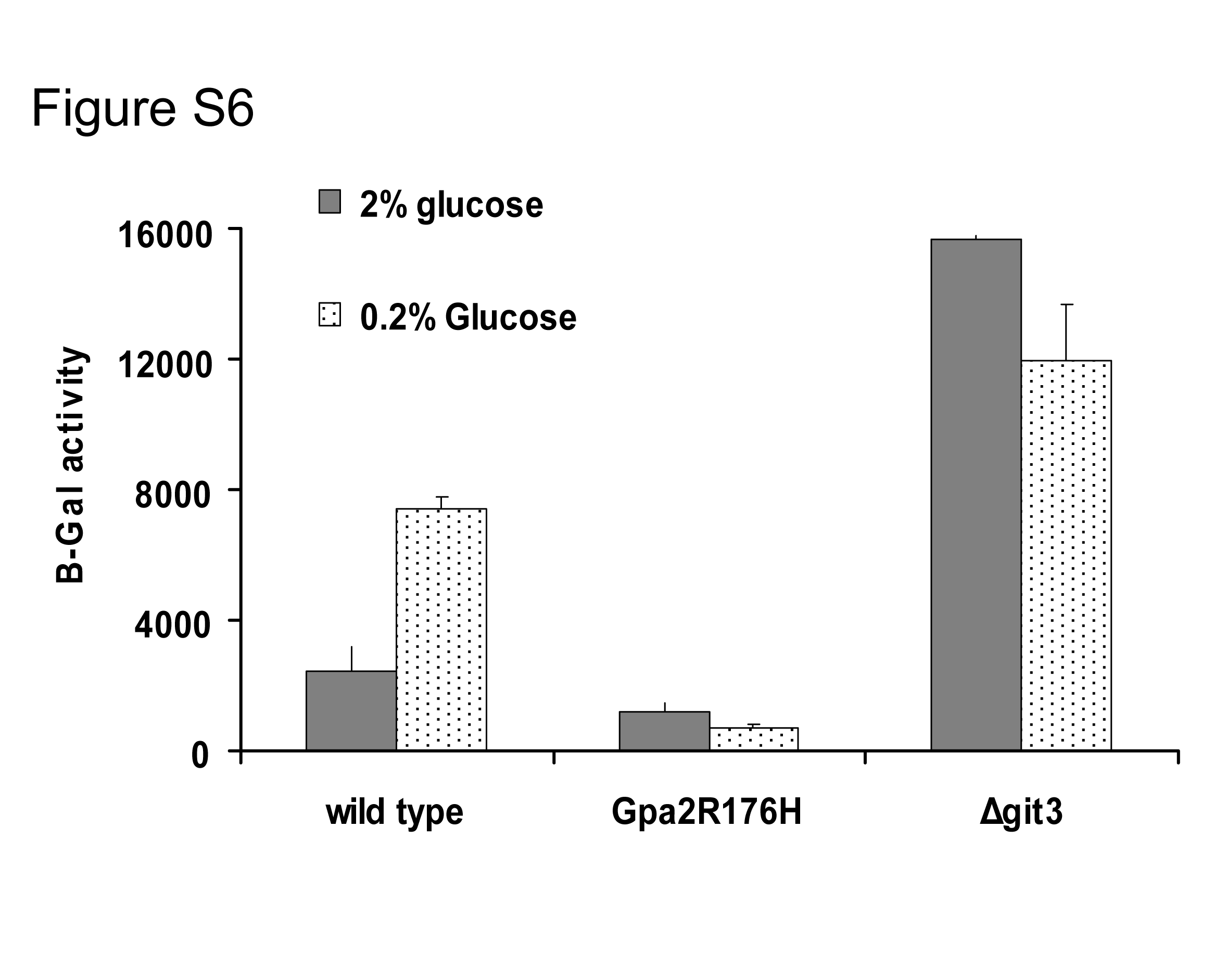

Supplement: Figure S6 — β-Galactosidase activity of Δgit3 and gpa2R176H with fbp1-lacZ reporter both grown in 2% and 0.2% glucose. Cells were collected at late exponential phase and β-Galactosidase activity was measured. (0.7 MB TIF) [file pgen.1000408.s006.tif]
