# Supplementary material for: Pro-Aging Effects of Glucose Signaling through a G Protein-Coupled Glucose Receptor in Fission Yeast
Source: PLoS Genet. 2009 Mar 6;5(3):e1000408. doi: 10.1371/journal.pgen.1000408 (PMC2646135; doi:10.1371/journal.pgen.1000408)
Supplement: Table S1 — Strains used in this study presented with their genotypes and the laboratory where they were created. * refers to strains created for this study. (0.03 Mb DOC) [file pgen.1000408.s007.doc]

| Strain | Genotype | Origin |
| --- | --- | --- |
| SP14000 (WT) | h^-^ *ade6-M210 leu1-32 ura4-D18 leu1-32* | Lab. stock |
| FWP87 | h*^+^ leu1-32 ura4::fbp1-lacZ fbp1::ura4^+^* | C. Hoffman |
| CHP984 | h^+^ *git3::Kan^R^* | C. Hoffman |
| RWP26 | h^+^ *ade6-M216 leu1-32 ura4::fbp1-lacZ git3::Kan^R^* | C. Hoffman |
| RWP1 | h^-^ *ade6-M216 leu1-32 ura4-D18 ura4::fbp1-lacZ gpa2-R176H* | C. Hoffman |
| RWP36 | h^+^ *ade6-M216 leu1-32 ura4::fbp1-lacZ gpa2-R176H* | C. Hoffman |
| CJM387 | h^-^ *ade6-M210 leu1-32 ura4-D18 his3-S18 hxk1::ura4^+^* | C. Gancedo |
| CJM389 | h^+^ *ade6-M216 leu1-32 ura4-D18 his3-D2 hxk2::his3^+^* | C. Gancedo |
| CHP1229 | h^+^ *ade6^-^ leu1-32 ura4::fbp1-lacZ his3^-^ hxk2::his3^+^* | C Hoffman^*^ |
| SP14383 | h^+^ *ade6-M216 leu1-32 ura4-D18 his3^-^ hxk1::ura4^+^ hxk2::his3^+^* | Lab stock^*^ |
| SP14405 | h^+^ *ade6-M216 leu1-32 ura4-D18 his3^-^ hxk1::ura4^+^ hxk2::his3^+^ pREP41_hxk2^+^* | Lab stock^*^ |
| SP14393 | h*^-^ leu1-32 ura4-D18 his3^-^ hxk1::ura4^+^ hxk2::his3^+^ git3::Kan^R^* | Lab stock^*^ |
| SP14412 | *h^-^ leu1-32 ura4-D18 his3^-^ hxk1::ura4^+^ hxk2::his3^+^ git3::Kan^R^* pREP41_*hxk2^+^* | Lab stock^*^ |
| SP14373 | *h^-^ leu1-32 ura4-D18 his3-S18 hxk1::ura4^+^ git3::Kan^R^* | Lab stock^*^ |
